# Positive outcome of average volume-assured pressure support mode of a Respironics V60 Ventilator in acute exacerbation of chronic obstructive pulmonary disease: a case report

**DOI:** 10.1186/1752-1947-6-284

**Published:** 2012-09-10

**Authors:** Miyuki Okuda, Makoto Kashio, Nobuya Tanaka, Takashi Fujii, Yoshinari Okuda

**Affiliations:** 1 Osaka Hospital, Neyagawakoen 2276-1, Neyagawa City, Osaka, 572-0854, Japan; 2 Okuda Clinic, Uchiage1123, Neyagawa, Osaka, 572, Japan

## Abstract

**Introduction:**

We were able to treat a patient with acute exacerbation of chronic obstructive pulmonary disease who also suffered from sleep-disordered breathing by using the average volume-assured pressure support mode of a Respironics V60 Ventilator (Philips Respironics: United States). This allows a target tidal volume to be set based on automatic changes in inspiratory positive airway pressure. This removed the need to change the noninvasive positive pressure ventilation settings during the day and during sleep. The Respironics V60 Ventilator, in the average volume-assured pressure support mode, was attached to our patient and improved and stabilized his sleep-related hypoventilation by automatically adjusting force to within an acceptable range.

**Case presentation:**

Our patient was a 74-year-old Japanese man who was hospitalized for treatment due to worsening of dyspnea and hypoxemia. He was diagnosed with acute exacerbation of chronic obstructive pulmonary disease and full-time biphasic positive airway pressure support ventilation was initiated. Our patient was temporarily provided with portable noninvasive positive pressure ventilation at night-time following an improvement in his condition, but his chronic obstructive pulmonary disease again worsened due to the recurrence of a respiratory infection. During the initial exacerbation, his tidal volume was significantly lower during sleep (378.9 ± 72.9mL) than while awake (446.5 ± 63.3mL). A ventilator that allows ventilation to be maintained by automatically adjusting the inspiratory force to within an acceptable range was attached in average volume-assured pressure support mode, improving his sleep-related hypoventilation, which is often associated with the use of the Respironics V60 Ventilator. Polysomnography performed while our patient was on noninvasive positive pressure ventilation revealed obstructive sleep apnea syndrome (apnea-hypopnea index = 14), suggesting that his chronic obstructive pulmonary disease was complicated by obstructive sleep apnea syndrome.

**Conclusion:**

In cases such as this, in which patients with severe acute respiratory failure requiring full-time noninvasive positive pressure ventilation therapy also show sleep-disordered breathing, different ventilator settings must be used for waking and sleeping. On such occasions, the Respironics V60 Ventilator, which is equipped with an average volume-assured pressure support mode, may be useful in improving gas exchange and may achieve good patient compliance, because that mode allows ventilation to be maintained by automatically adjusting the inspiratory force to within an acceptable range whenever ventilation falls below target levels.

## Introduction

**Figure 1:**
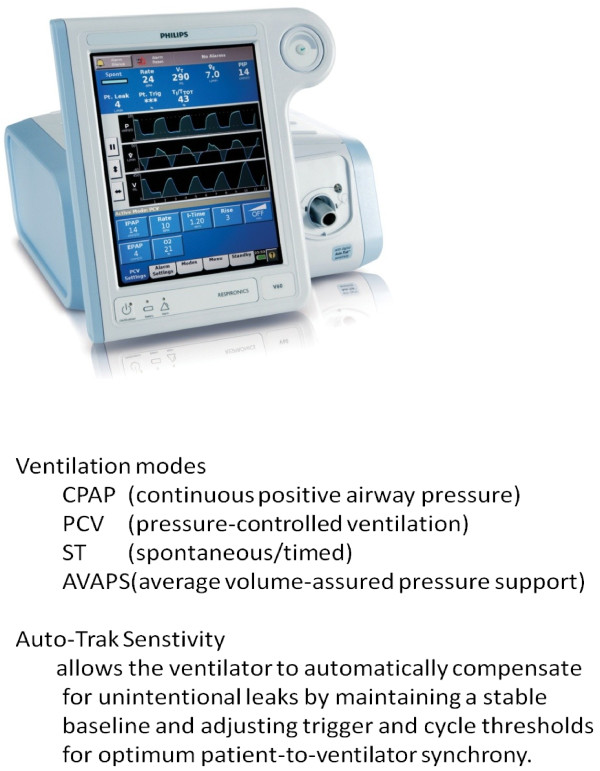
The Respironics V60 Ventilator.

The Respironics V60 Ventilator (Philips Respironics; United States) debuted in Europe and United States of America in 2009 and in Japan in 2010 as a successor model to the Vision Ventilator in providing biphasic positive airway pressure support ventilation (BiPAP) (Figure 1).

Pressure-controlled ventilation and average volume-assured pressure support (AVAPS) have been added as new ventilation modes to this model. We were able to treat a patient with acute exacerbation of chronic obstructive pulmonary disease (COPD), who also suffered from sleep-disordered breathing (SDB), without having to change the noninvasive positive pressure ventilation (NPPV) settings during the day and during sleep by using the AVAPS mode, which allows a target tidal volume to be set based on automatic changes in inspiratory positive airway pressure (IPAP).

## Case presentation

A 74-year-old Japanese man was being treated at home for type II respiratory failure due to COPD, but at the end of July 2011 he developed a fever of around 38°C and his dyspnea worsened. The oxygen flow rate was increased, but his hypoxemia failed to improve, and acute exacerbation of COPD was diagnosed on examination by his primary care physician. He was referred to our hospital and was hospitalized for further testing and treatment.

Our patient was 160cm tall, weighed 40kg, and had a temperature of 37.6°C. His heart rate was normal, at 110 beats/min, with an elevated respiratory rate of 28 breaths/min, blood pressure of 116/77mmHg, and oxygen saturation (SpO_2_) of 97% on nasal oxygen at 3L/min. Chest auscultation revealed bronchial rales in both his lower pulmonary fields. His white blood cell count was within the normal range, but his levels of C-reactive protein were highly elevated. Blood gas testing revealed pronounced hypercapnia (pH 7.292, partial pressure of oxygen (PaO_2_) 118.8mmHg, partial pressure of carbon dioxide (PaCO_2_) 81.5mmHg, bicarbonate (HCO_3_^-^) 38.2mmol/L, O_2_ intake 3L/min).

**Figure 2:**
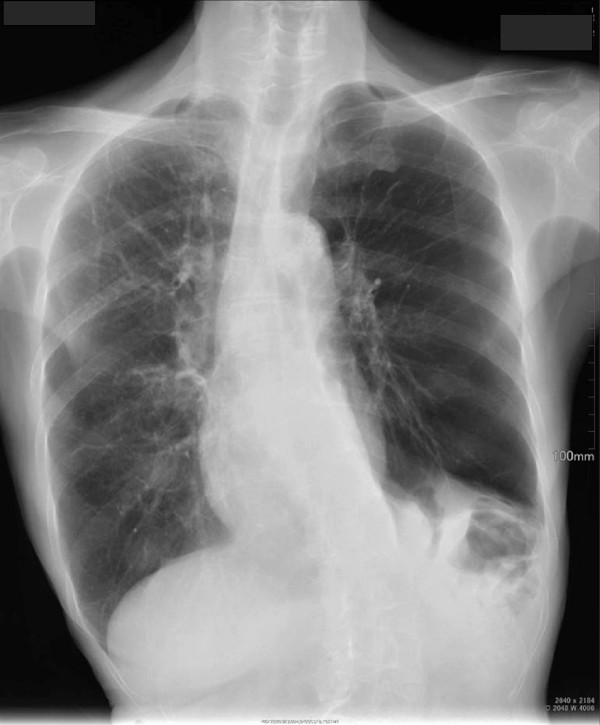
Chest roentgenography on admission showing multiple bullae and bilateral emphysema with left diaphragmatic hernia.

Radiography showed bilateral emphysematous lesions across all his pulmonary fields, with left diaphragmatic elevation and a right mediastinal shift (Figure 2).

**Figure 3:**
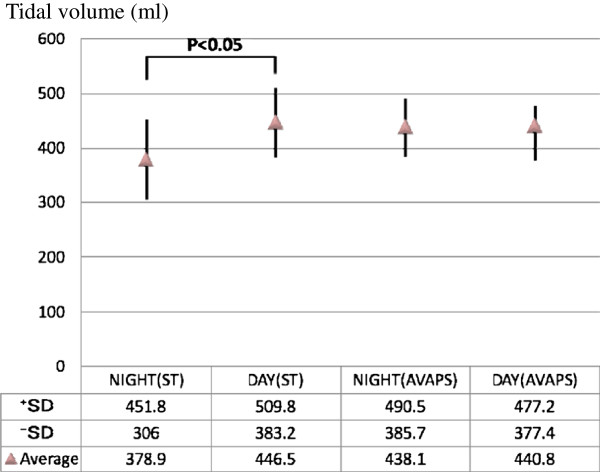
**Standard deviation indicating variance in tidal volume during spontaneous-timed and average volume-assured pressure support therapy.** ST: spontaneous/timed mode, AVAPS: average volume-assured pressure support, SD: spontaneous/timed mode.

Chest radiography and computed tomography showed no evidence of pneumonia, and he was diagnosed with acute exacerbation of COPD due to a respiratory tract infection on the basis of his clinical symptoms and sputum test results. He was placed on 24-h NPPV using a BiPap® Vision® Ventilatory Support System (IPAP, 8cmH_2_O; expiratory positive airway pressure (EPAP), 4cmH_2_O; spontaneous-timed **(**ST) mode), and was started on an antibiotic and a diuretic. As his respiratory status stabilized, he was taken off NPPV, which was then used only while sleeping at night, and his physical findings temporarily resolved. From around hospital day 32, however, a respiratory tract infection that may have been caused by aspiration triggered another episode of acute COPD exacerbation, with a depressed level of consciousness. This time, NPPV management was implemented using a Respironics V60 Ventilator (recently introduced at our institution) in AVAPS mode (IPAP, 8 to 10cmH_2_O; EPAP, 4cmH_2_O; target tidal volume, 350mL). Initial findings of pH 7.189, PaCO_2_ 136mmHg, PaO_2_ 108.1mmHg and HCO_3_^-^ 50.0mmol/L were indicative of hypercapnia and acidosis, and improved markedly to pH 7.508, PaCO_2_ 251.1mmHg, and PaO_2_ 75.9mmHg after one day on the V60 ventilator. NPPV was conducted using two different ventilators for his two episodes of acute COPD exacerbation. During BiPAP, his tidal volume was significantly decreased during sleep compared with waking hours, but use of the AVAPS mode of the Respironics V60 Ventilator automatically corrected the IPAP and was able to improve his sleep-related alveolar hypoventilation (Figure 3 and Additional file 1: Table S1). A polysomnography (PSG) was performed to diagnose our patient’s sleep respiratory disorder after his second episode of acute exacerbation.

The results of the PSG were as follows: sleep efficiency, 75.5%; apnea-hypopnea index, 14.0/h; apnea index, 2.7/h; hypopnea index, 11.3/h; mean nocturnal SpO_2_, 94%; minimum SpO_2_, 87%; SpO_2_<90% time (min) 15.0% with NPPV (ST with IPAP, 8cmH_2_O; EPAP, 4cmH_2_O; respiratory rate, 14/min; O_2,_ 1.5L/min).

## Discussion

Various lines of evidence have confirmed NPPV as useful for treating acute exacerbation of COPD [1-3]. The Global Initiative for Chronic Obstructive Lung Disease also recommends NPPV as the best option for effectively reducing the need for intubation, reducing nosocomial infections, primarily pneumonia associated with intubated mechanical ventilation, and reducing mortality [4]. The BiPap® Vision® Ventilatory Support System, which is generally used as an NPPV-dedicated device, was also used for the first episode of acute exacerbation in this case, and was able to improve the serious deterioration in respiratory status caused by the acute COPD exacerbation without intubated mechanical ventilation. According to the respiratory management records at that time, a significant decrease in his tidal volume was seen while our patient was asleep (378.9 ± 72.9mL) compared with his daytime tidal volume (446.5 ± 63.3mL) (Figure 3). The AVAPS mode of the Respironics V60 Ventilator was therefore used in an attempt to improve sleep-related hypoventilation during his second episode of acute COPD exacerbation. The Respironics V60 Ventilator, a successor model to the BiPap® Vision® Ventilatory Support System, comes with more sophisticated monitoring, an Auto-Trak™ system, and C-Flex™, as well as the addition of pressure-controlled ventilation and AVAPS ventilation modes. AVAPS is a new ventilation mode for pressure-support ventilation. The acceptable inspiratory range (IPAP(Max), IPAP(Min)) and target ventilation (Target V_TE_) are set, and respiration is monitored during ventilation, thereby allowing the ventilation to be maintained through automatic adjustment of the inspiratory force to within an acceptable range whenever the ventilation falls below the target level. Use of AVAPS has already been reported to have improved pronounced sleep-related decreases in oxygen saturation in a patient with congenital central hypoventilation syndrome [5]. This mode offers a greater improvement in ventilation efficiency during sleep, as compared with BiPAP, in patients with obesity hypoventilation syndrome resulting in significantly lower transcutaneous carbon dioxide [6]. A report of a randomized trial on the relationship between AVAPS and pressure support and improved sleep quality in nine patients with stable chronic hyperapnic COPD [7] concluded that, despite the short five-day period of use in the two modes in the study, AVAPS was as comfortable and effective as pressure support in alleviating respiratory acidosis, and produced better sleep efficiency than pressure support. We therefore used the V60 Ventilator in AVAPS mode in our patient, who exhibited sleep-related hypoventilation during the use of BiPAP. The ease with which IPAP settings for target ventilation are changed in AVAPS mode resulted in good patient compliance (his British Medical Research Council scale decreased to four from five) and allowed ventilation to be maintained by changing the settings from a mean daytime IPAP of 9.2cmH_2_O to a nocturnal IPAP of 9.6cmH_2_O. In fact, no significant difference was identified between daytime tidal volume (440.8 ± 36.4mL) and nocturnal tidal volume (438.1 ± 52.4mL) when AVAPS was used (Figure 3).

Various mechanisms are thought to contribute to the nocturnal hypoxemia that occurs with COPD [8]. First, COPD patients are exposed to hypoxemia while awake. Because this results in lower chemoresponsiveness of the respiratory center, nocturnal hypoxia or hypercarbia occur more readily. Second, muscular hypotonia of the skeletal muscles, including the respiratory muscles, occurs during sleep, particularly during rapid eye movement (REM) sleep. Respiration in patients with COPD is highly dependent on the accessory muscles of inspiration as opposed to the diaphragm, and this hypotonia during REM thus results in worsening of sleep-related alveolar hypoventilation. This, combined with factors such as decreased functional residual capacity, increases upper airway resistance and exacerbates ventilation-perfusion mismatch, resulting in profound hypoxemia, particularly during REM sleep. The possibility of nocturnal hypoxia in patients with COPD is therefore extremely important and requires appropriate management of ventilation during sleep. However, simply administering oxygen because of evidence of sleep hypoxia without taking hypoventilation and obstructive sleep apnea syndrome (OSAS) into consideration may lead to pronounced hypercapnia. Flenley included patients with this COPD (chronic bronchitis type) and sleep apnea syndrome (SAS) in the overlap syndrome category [9]_._ COPD is classified into the emphysema type (pink puffers) and the chronic bronchitis type (blue bloaters). In the latter patients in particular, advanced gas exchange disturbance and upper airway obstruction are believed to result in further exacerbation of hypoventilation during sleep and a pronounced tendency toward serious respiratory failure. A high percentage of patients with chronic bronchitis type are also thought to have OSAS, because such patients are often obese. The patient in our case had a very low body mass index of 15.6kg/m^2^ and was very thin, yet the PSG while our patient was on NPPV revealed SAS characterized by an apnea-hypopnea index of 14.0/h. Hypopnea-induced SDB predominated, at 11.3/h. These results are consistent with the report that the sleep hypoxia observed in patients with COPD is caused by hypopnea, whereas apnea is seldom observed [10]. OSAS is reportedly often more severe in Asians, considering that their body mass index is generally lower than in Westerners. This may be because of the more pronounced association of upper airway obstruction with smaller jaws and short necks [11]. The patient in our case also had a left diaphragmatic hernia, which may have exacerbated respiratory function failure in the supine position.

If measurement of nocturnal oxygen saturation reveals decreased oxygen saturation in patients with COPD, particularly those who also have pulmonary hypertension, PSG should be performed and appropriate treatment should be started. Simple administration of oxygen for sleep hypoxia can exacerbate hypercarbia at such times. Therefore, when upper airway obstruction predominates, some type of assisted ventilation will be needed for continuous positive airway pressure. In fact, in a study of patients with overlap syndrome not treated with CPAP (213 patients) compared with a group of patients with COPD alone (210 patients), the untreated group was associated with greater mortality (relative risk, 1.79; 95% confidence interval, 1.16-2.77) and acute exacerbations (relative risk, 1.70; 95% confidence interval, 1.21-2.38), but CPAP treatment was associated with improved survival (overlap patients with CPAP compared with the untreated overlap group, 7.5% versus 14.5% mortality, respectively) [12]. It has recently been recommended that NPPV should be used proactively as first-line treatment to improve not only acute exacerbations in patients with chronic respiratory failure, but also pulmonary edema due to acute cardiac failure [13]. European and Japanese guidelines recommend keeping SpO_2_ at 95% to 98% or more as a marker of oxygenation, but the large-scale ADHERE clinical trial on heart failure, which has enrolled more than 100,000 participants, revealed that as many as approximately 40% of patients fail to achieve SpO_2_ ≥ 95% [14]. Early introduction of NPPV is therefore a better option when routine oxygen administration fails to improve oxygenation. Patients with chronic heart failure often have SDB in the same manner as patients with chronic respiratory failure. Ferrier *et al.* reported that 53% of patients with heart failure have OSAS and that 13% have central SAS [15]. It is thus essential, in patients with heart failure as well, to be mindful of the complication of SDB during acute exacerbation and to set up a ventilator.

## Conclusion

On such occasions, the Respironics V60 Ventilator, which is equipped with an AVAPS mode, may be useful in improving gas exchange and may achieve good patient compliance, because that mode allows ventilation to be maintained by automatically adjusting inspiratory force to within an acceptable range whenever ventilation falls below target levels.

## Consent

Written informed consent was obtained from the patient for publication of this case report and any accompanying images. A copy of the written consent is available for review by the Editor-in-Chief of this journal.

## Abbreviations

AVAPS, Average volume-assured pressure support; BiPAP, Biphasic positive airway pressure support ventilation; COPD, Chronic obstructive pulmonary disease; EPAP, Expiratory positive airway pressure; IPAP, Inspiratory positive airway pressure; NPPV, Noninvasive positive pressure ventilation; OSAS, Obstructive sleep apnea syndrome; PSG, Polysomnography; REM, Rapid eye movement; SAS, Sleep apnea syndrome; SDB, Sleep-disordered breathing.

## Competing interests

The authors declare that have no competing interests.

## Authors’ contributions

MO and YO gathered the information for this case and were major contributors in writing the manuscript. MK, NT and TF contributed in writing the Discussion and editing the manuscript. All authors read and approved the final version of the manuscript.

## Supplementary Material

Additional file 1 **Table S1. **Measurements during spontaneous-timed and average volume-assured pressure support therapy. Click here for file
