# Supplementary material for: Positive outcome of average volume-assured pressure support mode of a Respironics V60 Ventilator in acute exacerbation of chronic obstructive pulmonary disease: a case report
Source: J Med Case Rep. 2012 Sep 10;6:284. doi: 10.1186/1752-1947-6-284 (PMC3485098; doi:10.1186/1752-1947-6-284)
Supplement: Additional file 1 — Table S1. Measurements during spontaneous-timed and average volume-assured pressure support therapy. [file 1752-1947-6-284-S1.docx]

|  | **Average** | **Standard Deviation** | **Maximum** | **Minimum** | **Number** |
| --- | --- | --- | --- | --- | --- |
| Tidal volume (ml)  Night (ST)  Day (ST)  Night (AVAPS)  Day (AVAPS) | 425.9  378.9  **＊**  446.5  438.1  440.8 | 64.1  72.9  63.3  52.4  36.4 | 571  480  571  540  505 | 279  279  361  350  415 | 35  9  11  10  5 |
| Minute volume (L)  Night (ST)  Day (ST)  Night (AVAPS)  Day (AVAPS) | 11.7  11.9  13.4  10.6  10.3 | 3.2  3.2  3.6  2.5  2.1 | 17  19  15  14  10 | 7  7  9  8.1  8.9 | 35  9  11  10  5 |
| IPAP (cmH2O)  Night (ST)  Day (ST)  Night (AVAPS)  Day (AVAPS) | 9.0  8.7  8.7  9.6  9.2 | 0.75  0.71  0.79  0.52  0.45 | 10  10  10  10  10 | 8  8  8  9  9 | 35  9  11  10  5 |
| RespiratoryRate(/min)  Night (ST)  Day (ST)  Night (AVAPS)  Day (AVAPS) | 21.5  25.0  26.2  16.6  15.0 | 8.6  9.6  9.5  3.2  1.7 | 42  42  38  24  18 | 14  18  14  14  14 | 35  9  11  10  5 |

　　*:p<0.05

AVAPS: average volume-assured pressure support; ST: spontaneous-timed.
